# Supplementary material for: Analysis of Systemic Inflammatory Factors and Survival Outcomes in Endometrial Cancer Patients Staged I-III FIGO and Treated with Postoperative External Radiotherapy
Source: J Clin Med. 2020 May 12;9(5):1441. doi: 10.3390/jcm9051441 (PMC7291051; doi:10.3390/jcm9051441)
Supplement: Supplementary file 1 [file jcm-09-01441-s001.pdf]

Table S1. Characteristics of patients and tumors.

Patients' characteristics		All patients *n* = 155 (100%)	Institut Gustave Roussy *n* = 77 (49.7%)	Hospital Clínic *n* = 78 (50.3%)
Time of follow-up (months)	Mean (SD)	46.3 (23.7)	42.1 (25.8)	50.4 (20.5)
	Median (range)	46.5 (4.0-108)	40.0 (4.0-108.0)	50.4 (7.2-88.3)
Age at diagnosis (years)	Mean (SD)	62.3 (10.7)	65.2 (10.4)	65.6 (10.5)
	Median (range)	63.2 (27.9-98.9)	64.1 (27.9-98.9)	65.0 (41.0-90.0)
	< 65 years	96 (61.9%)	40 (51.9%)	34 (43.6%)
	≥ 65 years	59 (38.1%)	37 (48.1%)	44 (56.4%)
FIGO 2009 stage (%)	IA	24 (15.5%)	14 (18.2%)	10 (12.8%)
	IB	37 (23.9%)	19 (24.7%)	18 (23.1%)
	II	36 (23.2%)	16 (20.8%)	20 (25.6%)
	IIIA	16 (10.3%)	12 (15.6%)	4 (5.1%)
	IIIB	7 (4.5%)	3 (3.9%)	4 (5.1%)
	IIIC1	24 (15.5%)	9 (11.7%)	15 (19.2%)
	IIIC2	11 (7.1%)	4 (5.2%)	7 (9.0%)
FIGO 2009 stage III	No (stage I-II)	97 (62.6%)	49 (63.6%)	48 (61.5%)
	Yes (stage III)	58 (37.4%)	28 (36.4%)	30 (38.5%)
Histology	Endometrioid	95 (61.3%)	42 (54.5%)	53 (67.9%)
	Serous-papillary	24 (15.5%)	13 (16.9%)	11 (14.1%)
	Clear cell	20 (12.9%)	16 (20.8%)	4 (5.1%)
	Mucinous	5 (3.2%)	5 (6.5%)	0 (0%)
	Carcinosarcoma	11 (7.1%)	1 (1.3%)	10 (12.8%)
Endometrioid histology	No	60 (38.7%)	35 (45.5%)	25 (32.1%)
	Yes	95 (61.3%)	42 (54.5%)	53 (67.9%)
Tumour grade	1	24 (15.5%)	15 (19.5%)	9 (11.6%)
	2	35 (22.6%)	15 (19.5%)	20 (25.6%)
	3	96 (61.9%)	47 (61.0.%)	49 (62.8%)
Tumour grade 3	No (grade 1-2)	59 (38.1%)	30 (39.0%)	29 (37.2%)
	Yes (grade 3)	96 (61.9%)	47 (61.0%)	49 (62.8%)
ESMO-ESGO-ESTRO 2015 Risk Assessment groups	3	13 (8.4%)	10 (13.0%)	3 (3.8%)
	4	134 (86.5%)	63 (81.8%)	71 (91.0%)
	5	8 (5.2%)	4 (5.2 %)	4 (5.1%)
Status at the end of data collection -Alive	Alive	115 (74.2%)	61 (79.2%)	54 (69.2%)
	Alive-disease free	113 (72.9%)	61 (79.2%)	52 (66.7%)
	Alive-in progression	2 (1.3%)	0 (0%)	2 (2.6%)
-Dead	Dead (any cause)	40 (25.8%)	16 (20.8%)	24 (30.8%)
	Cancer-related deaths	35 (22.6%)	14 18.2%)	21 (26.9%)
	Non cancer-related deaths	5 (3.2%)	2 (2.6)	3 (3.8%)
Time from surgery to cancer progression (months)	Patients with local or distant progression (%)	47 (30.3%)	23 (29.9%)	24 (30.8%)
	Media (SD)	17.8 (13.6)	16.3 (11.7)	19.3 (13.3)
	Median (range)	13.0 (0.66-72.5)	14.1 (4.0-72.5)	15.1 (0.66-51.3)

SD: standard deviation, IGR: Institute Gustave Roussy.

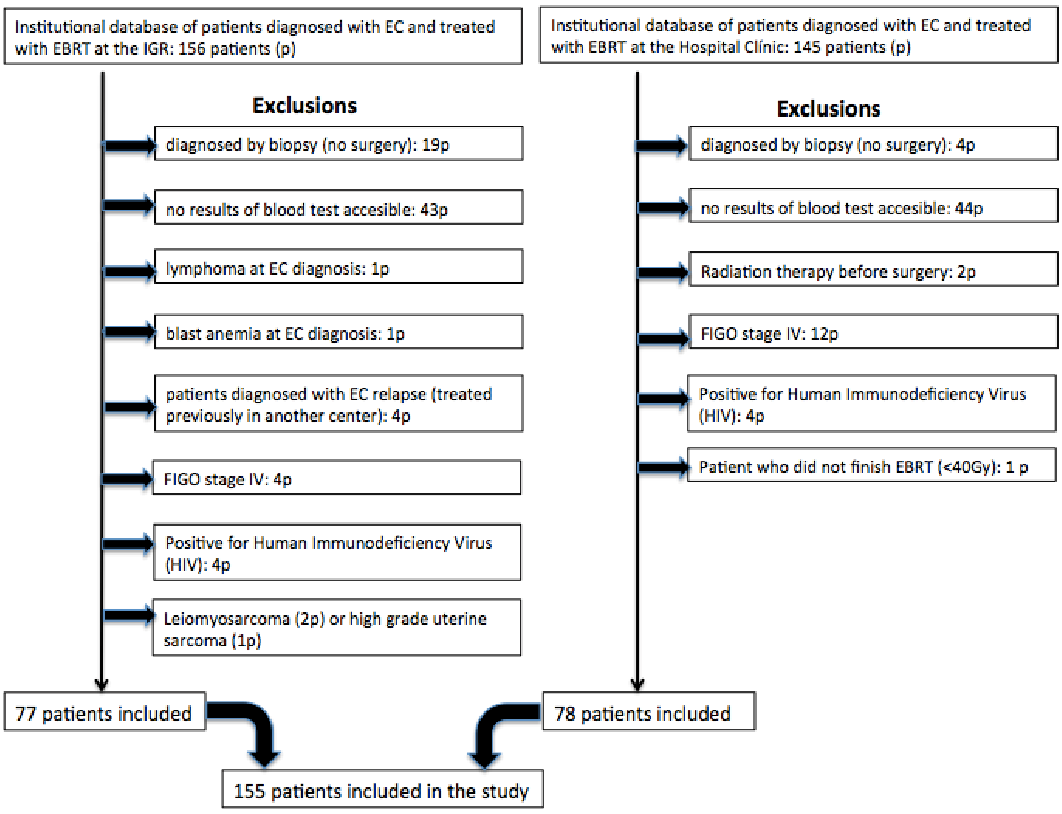

Figure S1. Exclusions flowchart (n = 155). EC: Endometrial cancer, EBRT: External Beam Radiotherapy, IGR: Institut Gustave Roussy, FIGO: International Federation of Gynecology and Obstetrics.
